# Fourth Disease

**Published:** 1902-02-15

**Authors:** 


### FOURTH DISEASE.

A very careful review of the question whether there really is what has lately been spoken of as a "fourth disease" has been made by Dr. Claude B. Ker,[1] medical superintendent of the Edinburgh City Hospital for Infectious Disease. Everybody, he says, nowadays admits that there is a third disease, usually called German measles, but spoken of also as rubeola, rubella, rötheln, and epidemic roseola; but rather more than a year ago Dr. Clement Dukes declared that he had discovered a "fourth disease" which had previously been included under one of the names given above, and the question is how far this claim can be substantiated. After discussing all the ins and outs of this question, Dr. Ker says that while he would not go so far as to say that a "fourth disease" does not exist, he would submit that in the meantime the evidence brought forward by Dr. Dukes is not sufficiently convincing. When Dr. Dukes published his paper Dr. Ker accepted his conclusions provisionally, and endeavoured, with the large amount of material at his disposal, to find facts in support of his claims. Now, however, after 18 months, he feels that only one verdict is possible in regard to this "fourth disease," and that is the Scottish one of "not proven."

Dr. Weaver,[2] on the other hand, again urges the specific nature of the disease, and appeals to his clinical experience in regard to 13 cases which occurred in 1900 and 1901 in the Southport Fever Hospital. Of these cases, seven were diagnosed as scarlet fever by medical practitioners in the town and sent into hospital as such. They there all developed scarlet fever after having apparently recovered from the complaint from which they were suffering when admitted. The remaining six were cases which were admitted into the hospital as scarlet fever, and when convalescent from that disease, developed "fourth disease," the infection having been caught no doubt from some of the other cases already referred to as having been inadvertently admitted into the wards among the scarlet fever cases. This certainly goes far to show that there may be such a separate disease as has been described, but when one comes to the practical side of the question, namely, that of diagnosis, we do not seem to get much further for Dr. Weaver says that even in a well-marked case the symptoms are those of a mild attack of scarlet fever with the difference that in "fourth disease" the rash (identical in other respects with that of scarlet fever) is out of proportion in intensity and extent to the other symptoms, while in other less typical cases it is quite impossible to distinguish in the early stages some of these cases of "fourth disease" from mild scarlet fever, and in such cases it is only by after-results, where they occur, that they are to be distinguished. All this is interesting, but in view of the wide divergences between different groups of cases of rubella and the possibility that these cases may have been examples of the scarlatiniform type of this disease it seems hardly convincing as a proof of the existence of a "fourth disease."

[1] Practitioner, Feb. [2] Brit. Med. Journ. Feb. 8.

